# Lateral photovoltaic effect in flexible free-standing reduced graphene oxide film for self-powered position-sensitive detection

**DOI:** 10.1038/srep33525

**Published:** 2016-09-16

**Authors:** In Kyu Moon, Bugeun Ki, Seonno Yoon, Jungwoo Oh

**Affiliations:** 1 Yonsei Institute of Convergence Technology, Yonsei University, Yeonsu-gu, Incheon 21983, Republic of Korea; 2 School of Integrated Technology, Yonsei University, Yeonsu-gu, Incheon 21983, Republic of Korea

## Abstract

Lightweight, simple and flexible self-powered photodetectors are urgently required for the development and application of advanced optical systems for the future of wearable electronic technology. Here, using a low-temperature reduction process, we report a chemical approach for producing freestanding monolithic reduced graphene oxide papers with different gradients of the carbon/oxygen concentration ratio. We also demonstrate a novel type of freestanding monolithic reduced graphene oxide self-powered photodetector based on a symmetrical metal–semiconductor–metal structure. Upon illumination by a 633-nm continuous wave laser, the lateral photovoltage is observed to vary linfearly with the laser position between two electrodes on the reduced graphene oxide surface. This result may suggest that the lateral photovoltaic effect in the reduced graphene oxide film originates from the built-in electric field by the combination of both the photothermal electric effect and the gradient of the oxygen-to-carbon composition. These results represent substantial progress toward novel, chemically synthesized graphene-based photosensors and suggest one-step integration of graphene-based optoelectronics in the future.

Ultralight-weight and inexpensive bendable films hold great promise for the development of next-generation flexible electronics, including eco-friendly wearable devices, (opto-)electronic skins, energy-storage devices, actuators and artificial muscles^1^,^2^,^3^,^4^,^5^,^6^. In particular, the present state of free-standing graphene oxide (GO) papers has emerged as a popular research areas area lately, focusing on improfving both highly electrically conductive and highly porous structures because of its flexibility and high mechanical strength^1^ such as energy storages^2^,^3^,^4^, conductors^5^, and environmental applications^6^,^7^. However, previous studies have not considered the potential effects of optoelectronic properties and applications. From nanoscale to microscale GO papers, these novel 2D materials are seldom considered as candidate materials for future flexible and wearable optoelectronic devices. Unfortunately, GO is not appropriate for use as an optoelectronic material due to the electrically insulating and intercalating properties of water molecules between the graphite oxide sheets. Consequently, further chemical combination of hydrazine vapor (*ex*., in the hydrazine liquid or gas phase) and high-temperature annealing processes are required to fabricate electrically conductive reduced GO (rGO) papers^8^. In particular, rGO-based nanomaterials have attracted attention due to the tunable optical and/or electrical properties in the different reduction levels of GO sheets^9^,^10^. Furthermore, both *p*- and *n*-type characteristics of rGO as a semiconductor have been reported; the *n*- or *p*-type character can be tuned as a function of the degree of reduction and/or doping levels of the rGO^11^,^12^. In view of these fascinating advantages, the fabrication of flexible rGO freestanding films is important for a variety of applications due to the low cost of low-temperature processing, as well as its mechanical flexibility, large area coverage, high reproducibility and ability to tune the topographical and electrical properties depending on the degree of reduction from GO, among other features. However, fabricating such a material is problematic due to its increased complexity as well as destroying their original high flexibility and integrity^13^,^14^, excluding polymer composites. Consequently, gas-phase reducing agents are extremely valuable for high-quality rGO paper. For example, Moon *et al.*^13^ have reported the synthesis of free-standing rGO film using hydrazine monohydrate vapor. However, this film is extremely fragile, prone to rupture and has a surface with a bumpy morphology. In comparison, hydriodic acid vapor is a more-efficient reducing agent for producing flexible rGO paper^13^. Presumably, hydriodic acid vapor molecules should be restricted to permeate between the internanosheets of GO paper. Thus, the “gradient-reduction depth” between the interior and exterior of the GO paper can be used to create different electrical and chemical properties along the direction of gravity. Both *p*-type and *n*-type interiors of rGO paper are roughly determined by the type of residual functional groups, such as electron-donating (EDG: *sp*^2^-bonded hydroxyl, ether and epoxy groups) or electron-withdrawing groups (EWG: carboxyl, carbonyl and *sp*^3^-bonded hydroxyl, ether and epoxide groups)^15^. Both the interior and the exterior-rGO internanosheets in rGO paper will give rise to unique electrical and optical properties when formed with different chemical compositions.

In this study, we present the simple design and preparation of a flexible micrometer-thick rGO membrane (∼1.4 μm thick) using hydriodic acid vapor at low temperature. This freestanding rGO membrane is a highly flexible, lightweight and electrically conductive three-dimensional foam with an open porous and interconnected graphene network. The sheet resistance of the resulting rGO membrane is less than ∼21–23 Ω·sq^−1^ and the conductivity is as high as ∼337.7–308.45 S·cm^−1^. Using the “gradient-reduction depth” concept mentioned above, we also explore the effect of lateral photovoltage on the freestanding self-powered GRAPHENE membrane-based metal–semiconductor–metal (MSM) photodetector, and possible operating mechanisms are proposed. This research advances promising strategies towards the design and fabrication of graphene-based composites in high-performance flexible optoelectronics, wearable electronics, and functional nanoscale devices.

## Experimental

### Preparation of the thick graphene oxide film (TGOF) and thick reduced graphene oxide (TrGOF) and device fabrication

The TGOF was produced by filtration of the GO dispersion solution (Graphene Supermarket Inc.) through an Anodisc membrane filter (4 mm in diameter, 0.2 μm pore size; Whatman), similar to the method reported for making GO paper^1^. TGOF thickness was tuned according to the volume of the GO dispersion solution. After the filtration, the as-prepared TGOF was air-dried for 2 days, before being peeled from the membrane filter. The TrGOF was reduced from the TGOF with hydriodic acid vapor using the Moon method^13^. The obtained TGOF onto Anodisc membrane filter was stand up Teflon slide tray (∼45° tilted angle) inside a 300 mL jar containing 0.5 mL of hydriodic acid (57 wt% in H_2_O) and 2 mL of acetic acid. The cover of the jar was sealed with vacuum grease and placed into an electric oven at 40 °C for 24 h. Subsequently, the TrGOF was carefully rinsed with a saturated sodium bicarbonate solution, water and methanol and dried in a vacuum oven at 50 °C for 24 h. The color of the TrGOF changed from reddish-brown to black with a metallic luster. The TrGOF was cut by operating scissors into rectangular strips of approximately 6 × 25 mm^2^ with an active length of 20 mm.

## General Methods

The UV-Vis spectra of the samples were recorded on a Varian Cary 5000 UV-Vis-NIR spectrophotometer. An atomic force microscopy (AFM) image of the TrGOF was obtained using an XE-100 (PSIA, Inc.) in noncontact mode. An X-ray diffraction (XRD) pattern was obtained using an X-ray diffractometer (SMARTLAB, Rigaku) equipped with conventional Cu Kα X-ray radiation. An Aramis Raman spectrum (HORIBA Jobin Yvon) using 514-nm laser excitation at room temperature was also obtained. X-ray photoelectron spectroscopy (XPS) was performed using a Thermo Fisher Scientific system (K-ALPHA ESCA SYSTEM) with a monochromatic Al Kα X-ray source. The XPS depth profile analysis was carried out using Ar ion sputtering and an X-ray probe (the etching rate was 0.1 nm·sec^−1^). Scanning electron microscope (SEM) and elemental mapping images were taken on a JEOL JSM-7100F. Water contact angle (WCA) measurements were performed to determine the wettability of the film surfaces using a Dataphysics Model OCA 15EC. The sheet resistance (R_sh_) value was measured using a four-point probe surface-resistivity meter (R-CHEK 4-pint sheet resistance meter, model RC2175). The sample thickness was obtained from SEM imaging of the fracture edge. The generated photocurrent was measured by a Keithley 2400 sourcemeter in a vacuum chamber equipped with a quartz window at room temperature. A 633-nm laser was mounted on a traveling microscope stage and its spot was moved along the length of the rGO membrane between the electrodes.

## Results and Discussion

**Figure 1:**
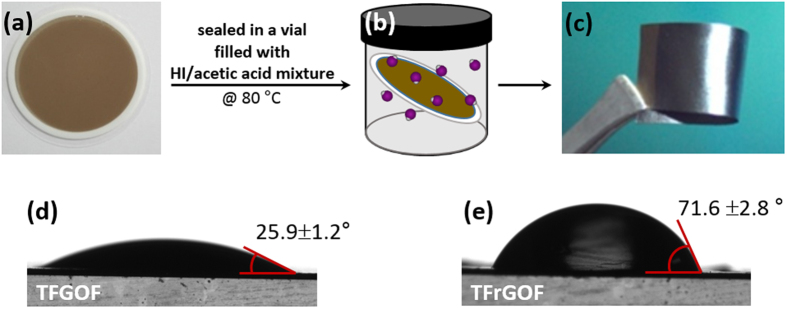
Fabrication process flow of the TrGOF. (**a**) A filtered TGOF on Anodisc membrane filter. (**b**) Reduced TGOF by hydriodic acid vapor. (**c**) Photograph of a TrGOF being thus demonstrating its flexibility. (**d**) Water contact angles and images of water droplets on the surface of TGOF (**d**) and TrGOF (**e**).

Figure 1(a,b) show schematic drawings illustrating the steps for fabrication of the TrGOFs. In a typical experiment, the mechanically flexible TGOFs are prepared by vacuum filtration of an aqueous GO dispersion (0.5 mg·mL^−1^)^1^. Afterwards, TrGOFs were reduced from TGOFs using HI in the gas phase at 80 °C according to the Moon method^13^. A photograph of a bent TrGOF is shown in Fig. 1(c), showing its good flexibility, as well as its smooth surface and shiny black color, which suggests the reduction of GO sheets. To achieve an in-depth insight into the surface characteristics of both the TGOF and the TrGOF, static contact angles were measured using water at room temperature (Fig. 1(d)). The water contact angles of the TGOF and TrGOF were 25.9° and 71.6°, respectively. Clearly, the TrGOF surface had a higher hydrophobicity, indicating that many oxygen atoms were removed by hydriodic acid vapor.

**Figure 2:**
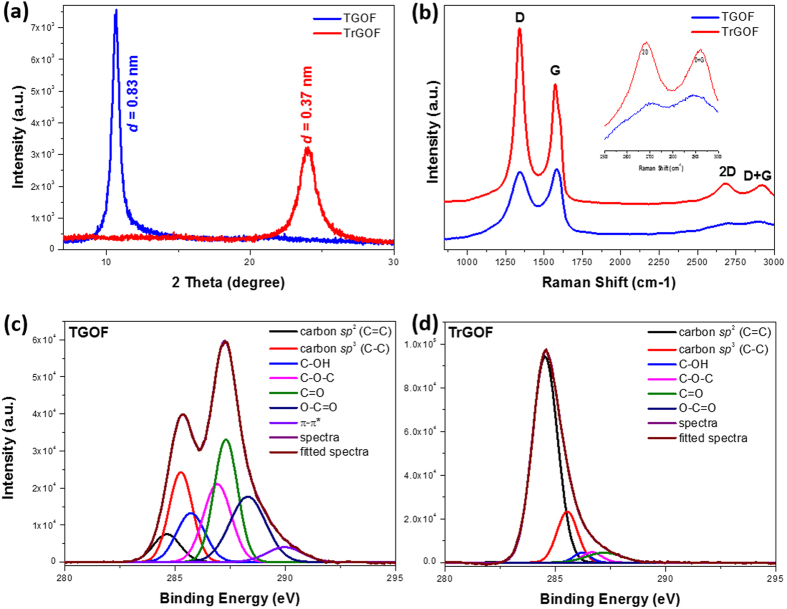
Characterization of the TrGOF. XRD (**a**) and Raman (**b**) spectra of TGOF and TrGOF. The inset in (**b**) is magnified neat the 2D and D + G bands. (**c**) High-resolution C1s XPS spectra of TGOF (**c**) and TrGOF (**d**).

To investigate the reduction of GO with hydriodic acid vapor, X-ray diffraction (XRD) spectroscopy was performed (Fig. 2(a)). The characteristic 2θ peak of TGOF appearing at 10.68° (full width at half maximum, FWHM = 0.68°) corresponds to a *d*-spacing of approximately 0.83 nm for the AB-stacked GOs, which is consistent with the interlayer spacing of GO sheets reported in the literature, because of the existence of the numerous oxygen functional groups on both sides/edges of the sheets and the water molecules trapped between them^16^,^17^. As expected, the TrGOF showed a distinct, broad peak at approximately 23.9° (FWHM = 1.54°) corresponding to a *d*-spacing of 0.37 nm, which indicates the removal of oxygen functional groups from GO sheets and the subsequent restoration of the *sp*^2^ carbon network by a hydriodic acid vapor-assisted process. This trend was also confirmed using Raman analysis, which was used to characterize the vibrational modes of the graphitic carbon lattice^18^,^19^. Raman spectroscopy is widely used in the structural characterization (crystal structure, disorder and defects, etc.) of carbon-based materials^20^. We used this information to verify the reduction process. Figure 2(b) compares the Raman spectrum of TGOF to that of TrGOF. Both TGOF and TrGOF have two distinct peaks: those at ∼1,340.9 and ∼1,574.1–1,579.5 cm^−1^ are the so-called D-band from defect-induced breathing modes of six-membered rings and G-bands from the first-order scattering of the E_2g_ phonon of *sp*^2^ C atoms using an excitation wavelength of 514.4 nm, respectively^20^. After reduction, the intensity of the D-band of TrGOF was increased compared to TGOF, indicating that the formation of more *sp*^2^ domains altered the structure of GO with a high-quality structural defects. The I_D_/I_G_ intensity ratio for TrGOF was larger than that for TGOF (∼1.34 for TrGOF and ∼0.97 for TGOF). This indicates that new graphitic domains were formed and that the smaller *sp*^2^ domain number also increased^13^,^21^ after the process described above, showing good reduction efficiency of the hydriodic acid vapor. The empirical Tuinstra-Koenig relation^22^, which is the ratio of the intensities of the D and G peaks, provides an indication of the degree of order across the graphitic planes. The *I*_D_/*I*_G_ ratio could also be used to determine the in-plane crystalline size (La) of the sample using the model given below:


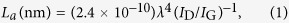


where λ(=514.5 nm) is the wavelength of the excitation laser line used in the Raman experiment. By applying the formula, the *L*_a_ of TrGOF was found to slightly decrease from 16.11 nm to 12.40 nm^23^. Furthermore, the 2D (∼2,681 cm^−1^) and D + G (∼2,919.6 cm^−1^) peaks of the as-prepared TrGOF appeared sharper than those of TGOF, strongly suggesting better graphitization and no charge transfer due to the absence of impurities in TrGOF^24^.

X-ray photoelectron spectroscopy (XPS) was used to study the reduction state of the GO sheet before and after the conversion process for better understanding of surface chemistries. Figure 2(c,d) provide the C 1s spectra of TGOF and TrGOF, respectively. The reduction effect is clear since the oxygen content of TrGOF is significantly reduced compared with that of TGOF. The components at 284.4 eV, 285.1 eV, 286 eV, 286.6 eV, 287.7 eV, 288.6 eV and 290.2 eV are related to *sp*^2^ C=C, *sp*^3^ C–C, C–OH, C–O–C, C=O, O–C=O and π–π^*^, respectively. Clearly, the peak intensities of the oxygen functional groups in TrGOF are lower than those in TGOF. The atomic ratio of carbon to oxygen (area of the C 1s peak divided by the area of the O 1s peak multiplied by the ratio of the photoionization cross sections) of TGOF and TrGOF are 1.69 and 4.94 (see Supplementary Figure S1), respectively, indicating the percentage of carbon in TrGOF that was graphitized by the *sp*^2^-hybrided carbon network during reduction. The C/O ratio of TrGOF decreased significantly compared to that of TGOF. In the O 1s spectra (see Supplementary Figure S1a,b), the peak at 533.1 eV was assigned to C–OH groups and the peak at 531.2 eV was assigned to C–O–C and C(O)OH groups^25^. These results suggest significant removal of oxygen functional groups.

**Figure 3:**
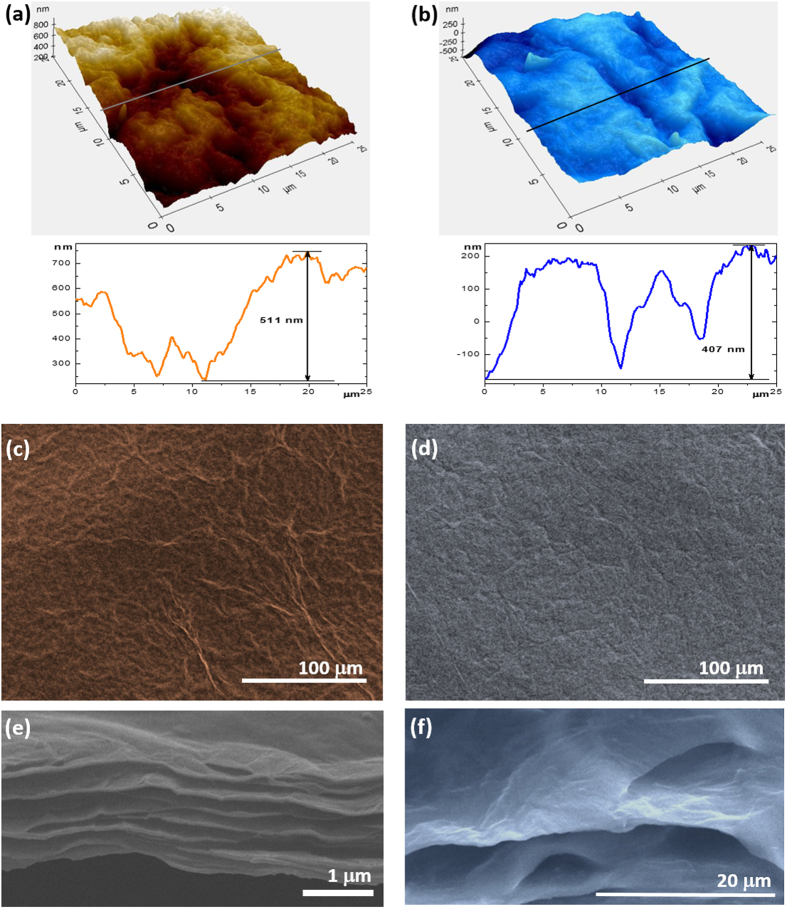
Micro-morphological characterization of TrGOF. AFM Stereographic plot of 25 × 25 mm^2^ images and corresponding profiles across the lines marked in the AFM images of TGOF (**a**) and TrGOF (**b**), respectively. SEM images of the surface morphology of (**c**) TGOF and (**d**) TrGOF. Side ((**e**,**f**) at higher magnification) of the cross-section morphologies of TrGOF.

Figure 3(a,b) show the AFM 3D pictures and surface profiles across the line marked in the AFM images of TGOF and TrGOF. The calculated root-mean-square surface roughness (the maximum peak-to-valley height) over an area of 25 × 25 μm^2^ was found to be 113 nm (0.611 μm) for the TGOF and 110 nm (0.407 μm) for the TrGOF, respectively. Top-view SEM images of both the TGOF and the TrGOF are shown in Fig. 3(c,d), and appear to have similar surface morphologies, namely corrugated wavelike formations. Corroboratively, the fracture edges of TGOF sample revealed well-packed layer structure under high-resolution scanning electron microscopy (SEM) (Fig. 3(c,d)). The rGO platelets were stacked tightly in the direction parallel to the thinner lamellae with interconnected porous structures within the TrGOF. Thinner lamellae had a thickness of about 34–80 nm, which corresponds approximately to ∼51–120 layers of rGO nanosheets. These thinner inter-lamellar spacings seemed to anchor neighboring features, making it obvious that these structures are highly interconnected in striation-like formations with air-pockets. The edge of one rGO platelet was entirely in contact with the neighboring platelet; thus, the platelets were effectively interlinked. It is believed that these features of highly-interconnected rGO lamellae within TrGOF induce conformational flexibility due to the “superlamellae” structure. The TrGOF had an average thickness of 1.41 μm including porous structures, leading to a lightweight architecture. The slow release and escape of the oxygen-functional groups of GO sheet reduced the distance between the rGO layers, thereby minimizing the volume. These morphological features clearly show great promise for applications in the field of electrodes for energy storage and selective absorption.

The measured sheet resistance (R_sh_) of TrGOF, obtained using a four-probe technology, was ∼21–23 Ω·sq^−1^, indicating sufficient reduction of TGOF by the hydriodic acid vapor. This value of R_sh_ for TrGOF is comparable to that of the hydrazine-mediated gas-phase reduction of a thick GO film (R_sh_ ∼338 Ω·sq^−1^)^13^. The low R_sh_ of TrGOF is consistent with the highest degree of deoxygenation and graphitization. The electrical conductivity of TrGOF was measured as ∼337.72–308.35 S·cm^−1^. The effect produced by UV was related to the behavior of the oxygen groups and their variation in content. It is also remarkable that the optical band gap of GO was around 3.06 eV and that the film thickness had nearly no effect on the optical band of GO^26^. Nevertheless, it may also be interesting to control the position and concentration of oxygen groups to enhance the optoelectronic properties of graphene, which are affected by the oxygenation of the surface. To further verify the high quality of the reduction of GO, we also characterized the UV-vis-near infrared (UV-vis-NIR) absorption spectrum of as-made thin rGO film on quartz (Supplementary Figure S2). The absorption maximum of the thin rGO film was ∼299 nm. This finding indicated that the electronic π-conjugation within the graphene sheets and the pronounced absorption extending to the NIR spectral region was restored upon reduction^27^, thus, this material has the potential for use in NIR optoelectronic devices. Furthermore, when the remaining oxygen functional groups on the rGO sheet had a larger electro-negativity than graphene, rGO became a *p*-doped material since the charge flow created negatively-charged oxygen ions and a positively charged carbon grid^28^,^29^.

**Figure 4:**
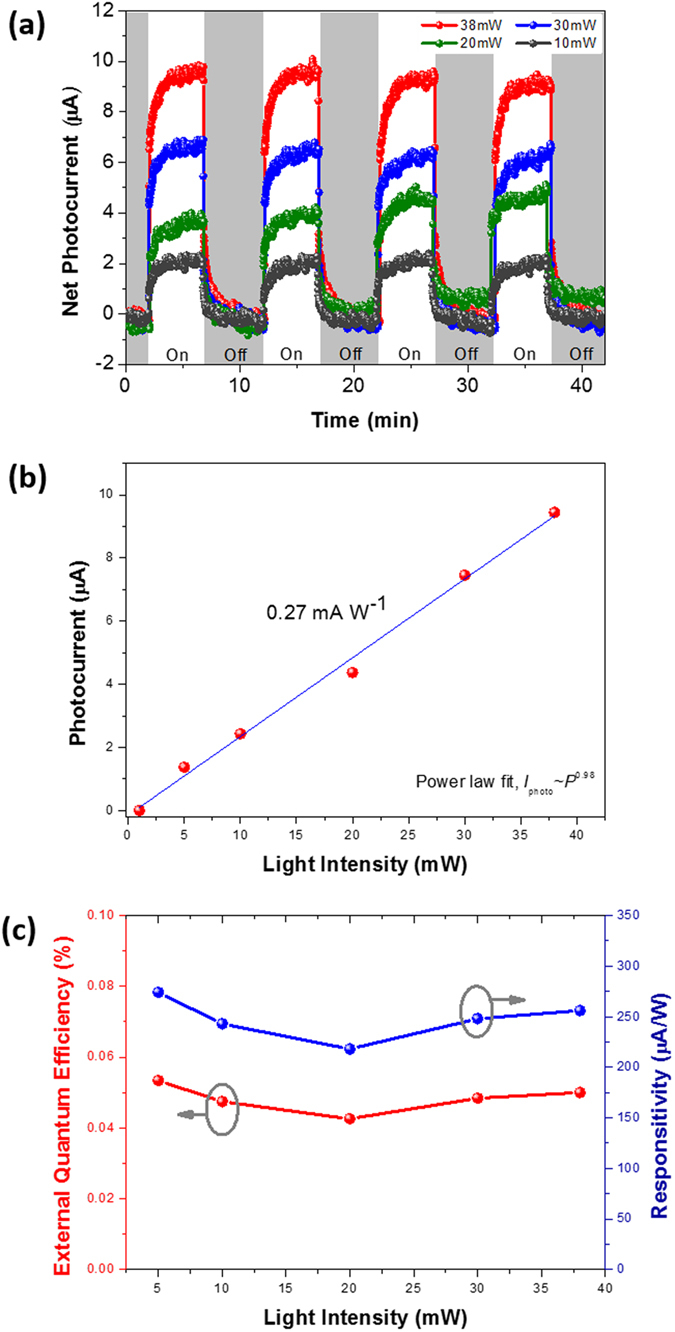
Photoresponse of the silver-TrGOF-silver structure under zero bias when the laser irradiated a region located 9 mm from center position. (**a**) Dynamic photocurrent-time curves as the laser was switched on and off under different light powers. (**b**) The linear fitting curve of saturation photocurrent versus light intensity, the slope of which shows the responsivity (about 0.27 mA·W^−1^) and (**c**) their relative responsivity and external quantum efficiency.

In order to obtain as flexible an optoelectronic paper as possible, the two ends of the TrGOF sample (∼6 ×25 cm^2^ and thickness ∼60–80 μm) were painted with silver (distance of between Ag electrodes∼ 20 mm), and the TrGOF fixed on a glass slide was pasted across two copper wire electrodes. To study the lateral photovoltaic effect in the TrGOF-based symmetric metal–semiconductor–metal (MSM) structure, we recorded the real-time photocurrent response as a function of the incident optical pump power at different laser-spot locations under zero set bias at room temperature under vacuum conditions (∼10^−3^ Torr). The corresponding data in Fig. 4(a) showed that the four repeat ON/OFF cycles of illumination were observed to be consistent and repeatable on a right-side position (+9 mm from center position (*x* = 0)) with different laser power levels. According to the previous reports, self-powered MSM photodetectors always require a different materials by cross the *p*-*n* junctions or asymmetric electrodes in MSM and/or traditional MSM photodetectors always require an external power source as the driving force to generate photocurrent due to their two symmetric Schottky contacts^30^. However, our device, with its large photoactive area, was different from that in ref. 30. Here, we need a slightly different approach to understand the physicochemical phenomena, as will be discussed later. The curve in Fig. 4(b) was fitted by a simple power law, I_photo _= AP^*a*^, where A is a proportionality constant, P is the light intensity and a is the exponent (0.5 < *a *< 1). According to previous reports, the non-unity exponent may be caused by a complex process of electron-hole generation, recombination and trapping within the semiconductor^31^,^32^. Figure 4(b) plots the detected photocurrent (P_input_) as a function of the incident power obtained at zero bias voltage, where the external responsivity (I_photo_/P_input_) is ∼0.27 mA·W^−1^. In our TrGOF device, I_photo_ exhibited a nearly linear behavior when *a* ∼0.98, as shown in Fig. 4(b), indicating a highly efficient generation of carriers transferred from photons.

Photoresponse time was another key parameter and was also relevant in revealing the physical mechanism behind the device’s operation. From the enlarged growth and decay curve shown in Fig. 4(a) for the laser power of 38 mW, the growth and decay times for our device were measured to be ∼42.5 and ∼56.4 s, respectively. Response times of growth and decay are defined as the time interval to change from 10% to 90% of the total increase or decrease, respectively^33^. When the laser was switched on, the photocurrent exhibited a rapid rise, followed by a slower increase until saturation after significant time had passed under illumination. After we turned off the laser, the initial photocurrent exhibited a quick decay, after which the decay process slowed down significantly. The growth and decay processes of the photocurrent followed an empirical biexponential functional equation,


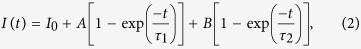


where *I*_0_ is defined as the steady-state photocurrent intensity under illumination, *A* and *B* are arbitrary constants, *t* is the time and *τ*_1_ and *τ*_2_ are the fast and slow time constants, respectively. The time constants for the biexponential photocurrent rise and decay were estimated to be *τ*_1,rise_ = 4.32 s, *τ*_2,rise_ = 73.98 s and *τ*_1,decay_ = 3.79 s, *τ*_2,decay_ = 70.02 s, respectively. The fast growth and decay times under illumination by 633-nm photons were attributed to a band-to-band transition that created conducting electrons and holes in the *sp*^2^ hybridization carbon atoms of graphene. The slow growth and decay processes could be attributed to the deep trap levels between the abundant oxygen-containing functional groups and the π–π interactions within the π-surface of the rGO sheets of the energy bandgap. Notwithstanding that the self-powered photoresponse speed was slower than that of chemical vapor-deposited graphene with a response time on the order of picoseconds^34^,^35^, the growth and decay times were comparable to, or shorter than, those of other graphenes and carbon nanotube composites^35^,^36^,^37^,^38^,^39^,^40^ under an *external bias*. Furthermore, the external quantum efficiency (EQE) can be calculated by EQE = (I_photo_/P_input_)·(*hc*/*e*λ), where *h* is the Planck constant, *e* is the electron charge, *c* is the velocity of light and λ is the wavelength of the incident light. The peak EQE in a TrGOF device was 0.05% under zero bias (Fig. 4(c)). The relatively low level of EQE was ascribed to the small effective absorption cross section of the TrGOF.

**Figure 5:**
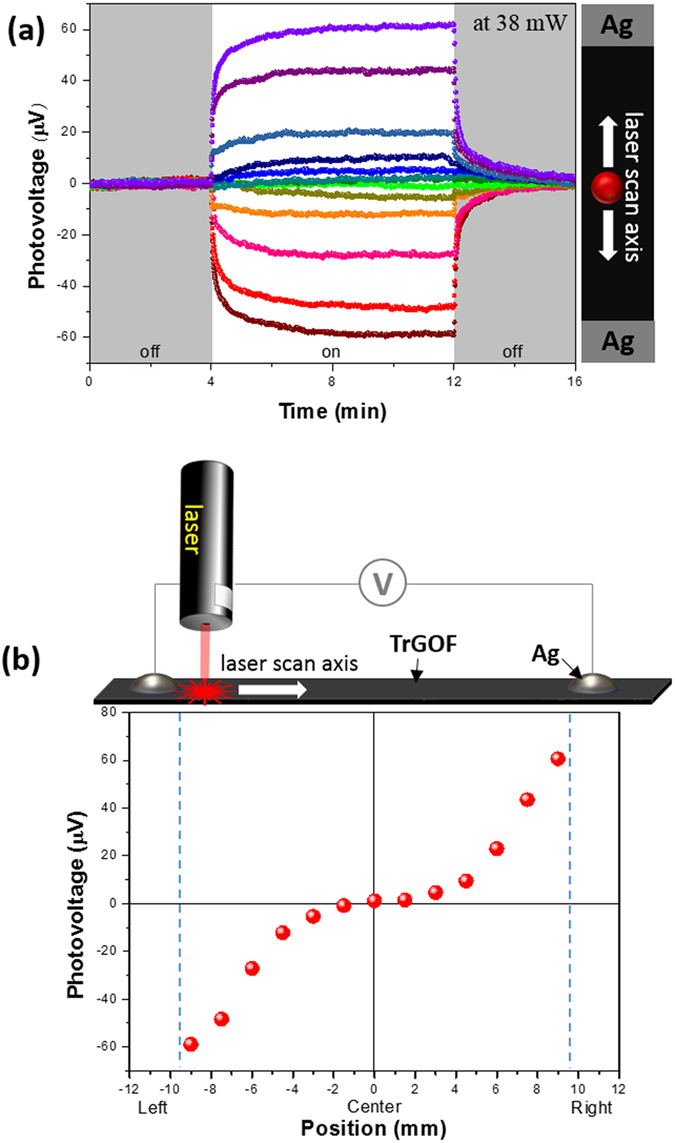
Lateral photovoltaic effect onto the silver-TrGOF-silver structure (**a**) The temporal photovoltaic response without any bias when laser spot irradiated region between electrodes. (**b**) The steady photovoltaic responses’ peak voltages depended nonlinearly on the laser spot positions through the zero point.

Another interesting observation is that the length of TrGOF’s channel responses analyzed the lateral photovoltage as a function of laser spot position as switching ON/OFF the laser illumination at a fixed light power of 38 mW (Fig. 5(a)). It is clear that, for each position, the lateral photovoltage sensitivities were always proportional to the light-exposure position on the TrGOF between the two electrodes. The lateral photovoltage peak is presented in Fig. 5(b) as a function of the laser spot position on the TrGOF between the two contacts. When the incident laser spot was at the center of the TrGOF between electrodes, the lateral photovoltage was almost zero due to the same lateral distance and diffusion symmetry. The lateral photovoltage showed the opposite charge polarity as the incident laser spot moved closer to the two electrodes. This nonlinearity of lateral photovoltage was reported in cases of long contact distance, higher resistivity^41^,^42^, and contact effects between the networked rGO nanosheets. In other words, the carrier density increased exponentially when the incident laser spot position was gradually changed from the center to each electrode. The maximum lateral photovoltage was observed when illuminating the region near the electrodes. As we moved the incident laser away from the electrodes, the lateral photovoltage dropped nonlinearly and disappeared at the center position between the two electrodes due to the smaller charge-diffusion length. This property makes self-powered photodetectors very useful in position-sensors for probing very small displacements and has a wide range of potential applications in a variety of fields, such as biomedicine, robotics, process control, position information systems and so on^43^.

**Figure 6:**
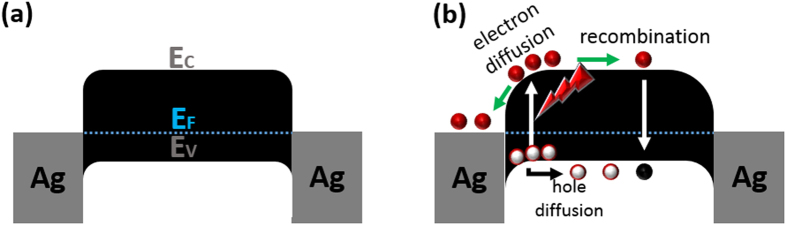
Energy band diagram of the Ag-TrGOF-Ag photodetector without (**a**) and with light (**b**).

**Figure 7:**
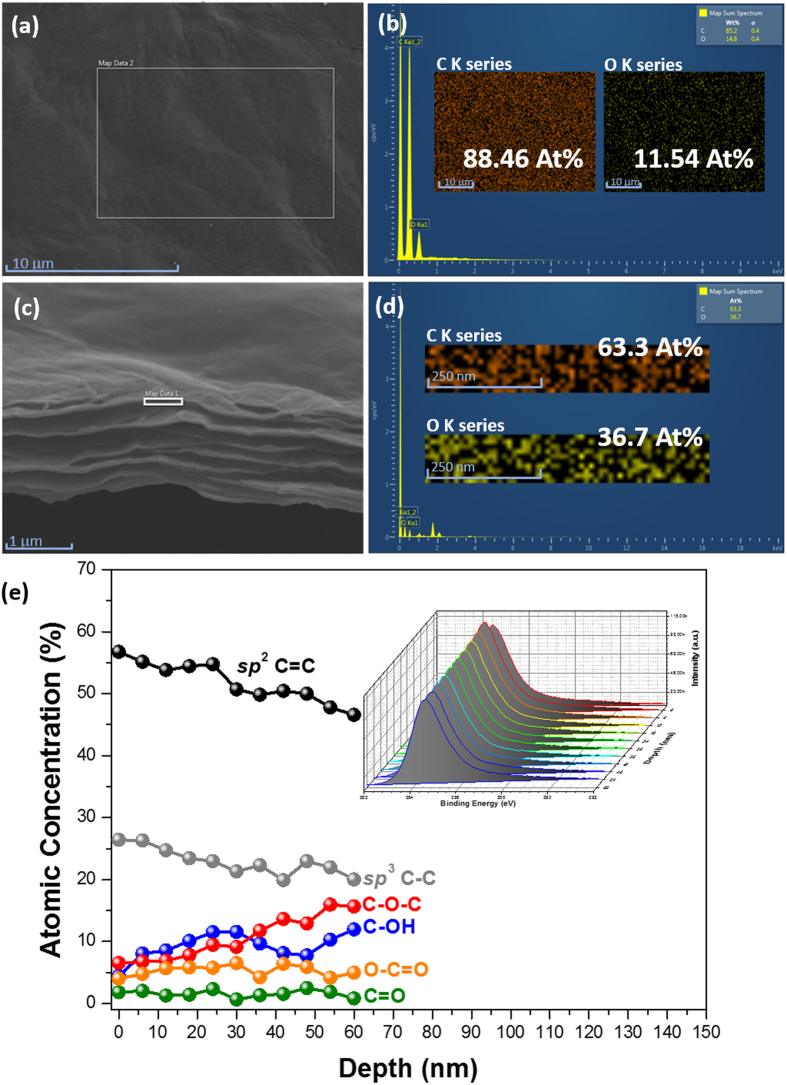
SEM image of (**a**) the surface image of the TrGOF, EDX spectrum (**b**) and corresponding element mapping (inset b). SEM image (**c**) of the cross section of TrGOF, EDX spectrum (**d**) and corresponding distribution of element (inset d). (**e**) Concentration depth profile of C 1s in the TrGOF using a monochromatic X-ray source and an argon ion-beam. Inset: the set of C 1s spectra measured during a depth profiling experiment.

Furthermore, the underlying physics of the lateral photovoltaic effects with spatially localized laser illumination can be understood according to band-diagram analysis, as illustrated in Fig. 6. With no illumination or drain voltage, the device in its equilibrium state is characterized by asymmetric Schottky barriers at graphene-electrode junctions^43^,^44^. Considering rGO as a *p*-type material with a Fermi energy greater than 4.5 eV^45^, which is larger than the Ag work function (4.26 eV), we plotted the schematic band diagram of the Ag-TrGOF-Ag device (Fig. 6). The photocurrent potential of TrGOF was always close to the Schottky barrier with a higher electrical potential. Lateral transport occurred due to hopping between localized states at the Fermi level in the *sp*^2^ sites. When the light is focused on the closer the Schottky contact under zero bias, the electron-hole pairs were generated by the photothermoelectric effect^5^,^46^,^47^ between the illuminated and the nonilluminated areas. In other word, the carrier mobility is much higher in the illuminated area, these major carriers have tendency to flow from the illuminated area to the nonilluminated region, which results in the formation of a transient electric field because the symmetry of the Seebeck coefficient across the junction was broken (Fig. 6). Upon light illumination, the generated electron-hole pairs are quickly swept away from this area in opposite directions by the Schottky contact induced localized electric field, which leads to the efficient collection of photocarriers at the metal electrodes due to the depressed recombination rate. The carriers could not return into the rGO film due to the Schottky barrier’s asymmetric profile (back-to-back Schottky contacts)^44^. And then, the gradually heterojunction barriers p-junction between rGO upper layer (the highly reduced GO) and rGO inner layer (the reduced gradient GO) in micro-thick TrGOF membrane (Figs 6 and 7) may be creates a lateral photovoltage gradient by the space charge region, which pushes both electrons and holes for enhanced diffusion. As a result of which, the electric potential is higher than the opposite electrode, the lateral photovoltage will form between these two electrodes. When, on the contrary, the light spot is focused at the center position between silver electrodes on the TrGOF, the photovoltage is zero due to the diffusion symmetry.

In order to confirm whether the *p*-junction in the rGO multilayers was gradually formed within the TrGOF membrane, X-ray energy dispersive spectroscopy (EDS) mappings of both the rGO upper layer and the rGO inner layer were collected in Fig. 7(a–d), respectively. An elemental mapping for carbon and oxygen, corresponding to the white square marked in the SEM images (Fig. 7(b,d)), indicated a uniform distribution of oxygen across both the rGO upper layer and the rGO inner layer of the TrGOF membrane. The results obtained show that the atomic percentage of carbon in the cross-section of the TrGOF membrane was much lower than in the surface of TrGOF membrane while the atomic percentage of oxygen in the cross-section of the TrGOF membrane was greater than that in the TrGOF membrane. As expected, analysis revealed higher levels of oxygen in the interior of the TrGOF membrane than at its surface. However, the EDS analysis was insufficiently accurate to provide the exact oxygen content of the TrGOF membrane. In order to further characterize the TrGOF membrane in detail, the XPS analysis depth from 0 to 60 nm was characterized. These XPS depth profiles provided the characteristic features of the distribution of elements in the rGO layer. The deconvoluted C 1s spectra of the TrGOF membrane showed peaks at 284.4 eV, 285.1 eV, 286 eV, 286.6 eV, 287.7 eV and 288.6 eV, which corresponded to the carbon atoms in graphite, *sp*^2^ C=C, *sp*^3^ C–C, C–OH, C–O–C, C=O and O–C=O, as shown in Fig. 7(e). This finding reveals that significant changes occurred in the relative ratios of the *sp*^2^ C=C and *sp*^3^ hybrid forms of carbon (C–C and various oxygen functionalities), indicating the degree of anisotropic reduction along the lateral cross section. Figure 7(e) shows the fitted peak area as a function of etching depth. From Fig. 7(e), it can be seen that the peak areas of *sp*^2^-bonded epoxide and *sp*^2^-hydroxyl groups as electron-donating groups tended to increase along with etching depth. On the one hand, *sp*^2^ C=C and *sp*^3^ C–C fractions decreased with increasing etching depth, while on the other, the fractions of *sp*^2^-carbonyl and *sp*^2^-carboxyl as electron-withdrawing groups remained almost unchanged. The results showed that the oxygen content of the TrGOF membrane increased from the surface to the bottom. The above-mentioned XPS depth-profile results confirmed the electron-donation between the *sp*^2^-bonded epoxide and the *sp*^2^-hydroxyl groups, as well as the fact that the gradient *p*-doping can be induced along the lateral section from the viewpoint of chemical composition. This discrepancy caused the accumulation of holes near the metal electrode in the rGO’s multilayer region; therefore, the Schottky barrier height of the rGO was reduced.

## Conclusions

In summary, we have prepared a flexible and lightweight porous rGO membrane using a simple chemical-vapor-reduction method based on the concept of the “gradient-reduction depth” of a graphene oxide membrane. Our method for producing freestanding rGO–based porous films is much simpler and more cost-effective than conventional methods and is well-suited to mass production. Moreover, we have also experimented with a flexible, large photoresponsive-active area and self-powered position-sensitive photodetector using a flexible freestanding graphene film on a symmetric MSM device structure. There is no need for an external power source for self-powered light sensing, as the device is powered by photothermal electricity generation. Our present results provide novel insights into the material design of a position-sensitive photothermoelectric detector without external power connections, and this flexible rGO porous film with an interconnected 3D network is not only suitable for energy storage (e.g., lithium-ion batteries or supercapacitors) but may also have other applications, such as oil-spill cleanup, gas-sensor platforms, and conducting substrates.

## Additional Information

**How to cite this article**: Moon, I. K. *et al.* Lateral photovoltaic effect in flexible free-standing reduced graphene oxide film for self-powered position-sensitive detection. *Sci. Rep.* **6**, 33525; doi: 10.1038/srep33525 (2016).

## Supplementary Material

Supplementary Information
