# Corrigendum: Lateral photovoltaic effect in flexible free-standing reduced graphene oxide film for self-powered position-sensitive detection

**DOI:** 10.1038/srep36800

**Published:** 2016-11-11

**Authors:** In Kyu Moon, Bugeun Ki, Seonno Yoon, Jongwan Choi, Jungwoo Oh

Scientific Reports 6: Article number: 3352510.1038/srep33525; published online: 09 16 2016; updated: 11 11 2016

Jongwan Choi was omitted from the author list in the original version of this Article. This has been corrected in the PDF and HTML versions of the Article, as well as the Supplementary Information file.

The author contributions section now reads:

“I.K.M. and J.O. designed the research and initiated the study. I.K.M., S.Y. B.K. and J.C. prepared and characterized the material. All authors contributed to the discussion”

