# Supplementary material for: Lateral photovoltaic effect in flexible free-standing reduced graphene oxide film for self-powered position-sensitive detection
Source: Sci Rep. 2016 Sep 16;6:33525. doi: 10.1038/srep33525 (PMC5025851; doi:10.1038/srep33525)
Supplement: Supplementary Information [file srep33525-s1.doc]

**Supplementary Information**

**Lateral photovoltaic effect in flexible free-standing reduced graphene oxide film for self-powered position-sensitive detection**

In Kyu Moon,^1^ Bugeun Ki,^1,2^ Seonno Yoon^1,2^ and Jungwoo Oh^1,2^

^1^Yonsei Institute of Convergence Technology, Yonsei University, Yeonsu-gu, Incheon 406-840, Republic of Korea and ^2^School of Integrated Technology and Yonsei Institute of Convergence Technology, Yonsei University, Yeonsu-gu, Incheon 406-840, Republic of Korea.

Correspondence: Professor J. Oh, ^2^School of Integrated Technology and Yonsei Institute of Convergence Technology, Yonsei University, Yeonsu-gu, Incheon 406-840, Republic of Korea.

E-mail: jungwoo.oh@yonsei.ac.kr


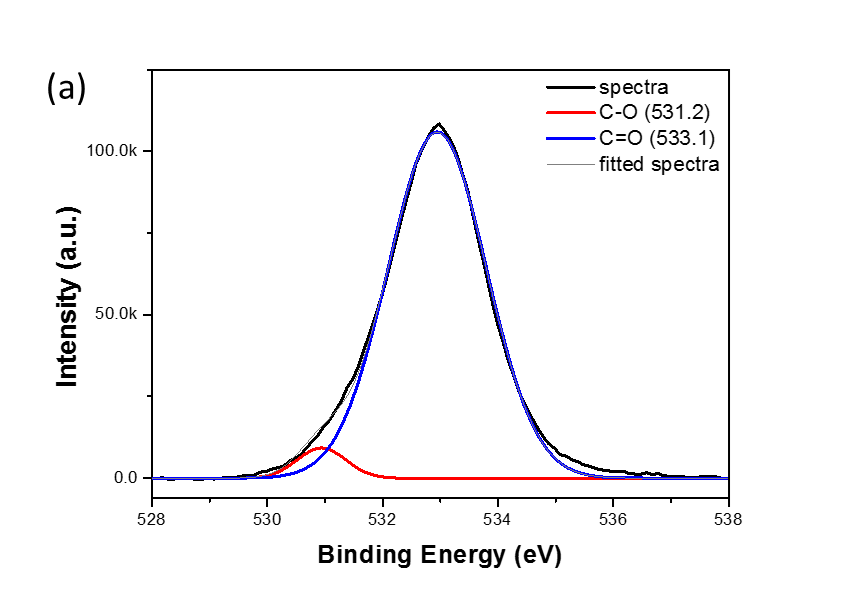


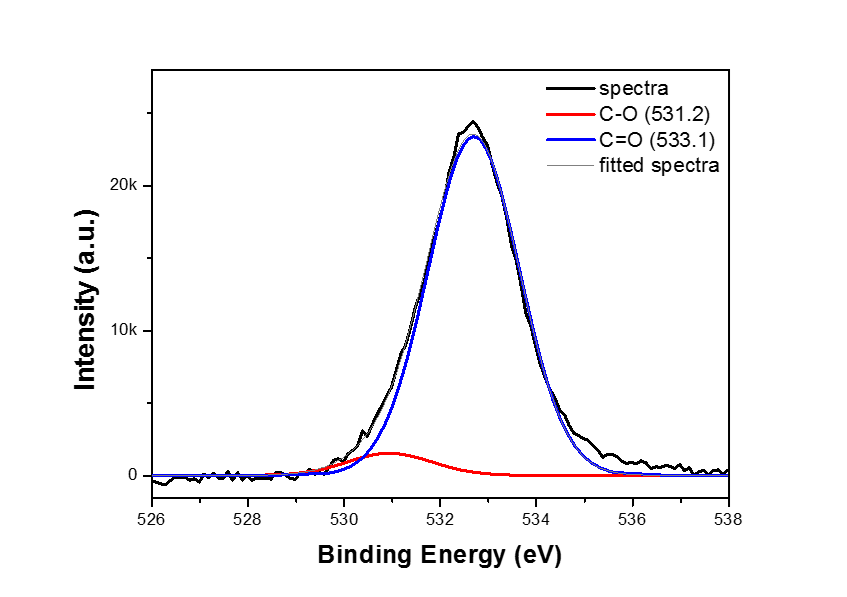


**Figure S1.** O 1s core level spectra of the TGOF(a) and TrGOF(b).

**Figure S2.** UV-vis-NIR adsorption spectrum of rGO film.
